# Strength and Durability of Respiratory Syncytial Virus Prefusion F Immunoglobulin G Following Infection and Exposure in a Household Cohort, 2014–2022

**DOI:** 10.1093/infdis/jiaf168

**Published:** 2025-04-03

**Authors:** Kalee E Rumfelt, Casey Juntila, Matthew Smith, Amy Callear, Yangyupei Yang, Arnold S Monto, Adam S Lauring, Emily T Martin

**Affiliations:** Department of Epidemiology, University of Michigan, Ann Arbor, MI, United States; Department of Epidemiology, University of Michigan, Ann Arbor, MI, United States; Department of Epidemiology, University of Michigan, Ann Arbor, MI, United States; Department of Epidemiology, University of Michigan, Ann Arbor, MI, United States; Department of Epidemiology, University of Michigan, Ann Arbor, MI, United States; Department of Epidemiology, University of Michigan, Ann Arbor, MI, United States; Division of Infectious Diseases, Department of Internal Medicine, University of Michigan, Ann Arbor, MI, United States; Department of Microbiology and Immunology, University of Michigan, Ann Arbor, MI, United States; Department of Epidemiology, University of Michigan, Ann Arbor, MI, United States

**Keywords:** antibody, households, humoral immunity, IgG, RSV

## Abstract

**Background:**

Little is known about the strength and durability of protection provided by prefusion F (pre-F) immunoglobulin G (IgG) after respiratory syncytial virus (RSV) infection/exposure.

**Methods:**

We analyzed 1019 sera from 422 individuals in 173 households, collected 365 days before and after RSV infection or exposure (2014–2022), from a longitudinal cohort with active respiratory infection surveillance. IgG against RSV pre-F was measured by electrochemiluminescence assay. We used a Cox model, adjusted for age, to assess the association between log_4_ preinfection/exposure IgG and risk of infection. We compared pre- and postinfection/exposure IgG geometric mean concentration increases among cases and household contacts to identify asymptomatic infections. Generalized additive mixed models predicted IgG concentrations over time.

**Results:**

We identified 113 confirmed RSV cases and 377 exposed household contacts. Cases had significantly lower pre-F IgG before infection (*P* < .05) and significantly higher levels after infection (*P* < .05). A 1-unit increase in log_4_ preinfection IgG decreased the risk of infection by 25% (*P* < .05). Among 58 cases with pre- to post-RSV geometric mean concentration increases, the mean fold increase was 1.12. Eight individuals without confirmed infections had ≥1.12-fold increases and were classified as probable asymptomatic infections. Cases had the highest IgG concentrations after infection, peaking at 1 month (*P* < .001).

**Conclusions:**

Pre-F IgG is a reliable correlate of protection for RSV infection risk. We found that RSV pre-F IgG–mediated protection starts to wane 6 months after infection. Therefore, scheduling of RSV vaccination should be evaluated so that individuals with the highest risk of severe disease are protected throughout the RSV season.

## Teaser

Prefusion F immunoglobulin G is a reliable respiratory syncytial virus (RSV) correlate of protection. We found that prefusion F immunoglobulin G–mediated protection starts waning 6 months after infection. Therefore, RSV vaccination scheduling should be evaluated so that individuals with the highest risk of severe disease are protected throughout the RSV season.

Respiratory syncytial virus (RSV) infects people of all ages but primarily causes severe disease in infants and the elderly [1]. Although symptomatic illness occurs more often in infants and older adults, all individuals are repeatedly exposed to and infected with RSV throughout their lifetime [2]. Older children and younger adults (approximately 12–50 years old) are more likely to have asymptomatic RSV infections and can experience multiple asymptomatic infections annually [3–6]. Therefore, households with young children are often exposed to RSV, and their exposed household contacts may experience symptomatic or asymptomatic illness. Measures that may predict the likelihood of RSV infection, also known as correlates of protection (CoPs), provide metrics for assessing infection risk [7]. CoPs have primarily been studied in relation to vaccine responses; however, a relative CoP can also be used to assess the continuum of risk associated with natural immunity.

RSV infection, vaccination, and immunotherapy provide protection against future infections via immunoglobulin G (IgG) antibodies toward the immunodominant prefusion F (pre-F) glycoprotein [8, 9]. While pre-F antibody function is not the only immunologic mechanism that prevents infection, levels of pre-F antibodies have been used as CoPs to estimate a person’s susceptibility to symptomatic RSV infection [9]. However, due to limited data, variations in testing, and low capture of asymptomatic cases, little is known regarding the strength and duration of protection that RSV pre-F antibodies confer after infection or exposure [7, 9, 10]. Specifically, there is variability in published information regarding how RSV pre-F IgG levels before infection or exposure relate to one’s susceptibility to subsequent RSV infection [3, 11–13]. In addition, there is a dearth of longitudinal data on IgG dynamics following RSV infection or exposure and an understanding of how these changes relate to RSV infection risk. The little information regarding IgG dynamics following infection or exposure that is available is primarily limited to clinical settings assessing monoclonal antibody therapy, which does not provide an accurate representation of RSV IgG dynamics in real-world settings [14].

With the ongoing development of RSV vaccines and monoclonal antibodies, much is being learned about the durability of RSV immunity in certain populations. However, it is important to have a better understanding of RSV antibody dynamics, particularly the durability of antibody responses, over multiyear periods in observational settings where RSV vaccines and monoclonal antibodies are now being applied. Here, we utilized 7 years of data from a longitudinal household cohort study in Michigan. The use of household data is advantageous for this research question because it allows for temporal evaluation of antibody levels among people infected with RSV and their exposed household contacts. We measured RSV pre-F IgG at 3 time points from individuals with confirmed RSV infections and their exposed household contacts. The IgG concentrations were used to determine (1) the level of IgG antibody preceding the infection/exposure that correlated with protection, (2) the magnitude of antibody boosts following infection/exposure, and (3) the timing of antibody waning following infection/exposure in mild to moderate and asymptomatic infections in a community setting.

## METHODS

### Study Population

Specimens and data from individuals eligible for this analysis were obtained from the Household Influenza Vaccine Evaluation (HIVE) cohort between 2014 and 2021, which has been previously described [15]. Briefly, the HIVE cohort consists of households within 30 miles of the study clinic in Ann Arbor, Michigan, with at least 3 persons who receive primary care within the University of Michigan Health System and have at least 1 child aged <18 years living in the household. To be eligible for inclusion in the analysis, households must have had at least 1 RSV infection identified between 2014 and 2022, as confirmed by reverse transcription polymerase chain reaction (RT-PCR). This study was reviewed and approved by the institutional review board of the University of Michigan Medical School (HUM00034377, HUM00118900); the study was conducted in accordance with the ethical standards of the Helsinki Declaration.

### Data and Sample Collection

All information regarding data collection, acute respiratory infection (ARI) case definition, ARI sampling, and ARI testing has been described [15]. Briefly, a nasal swab was collected within 7 days of illness onset at the HIVE clinic by trained staff, and RT-PCR RSV testing was performed if a patient reported 2 ARI symptoms. All household members ≥18 years of age provided written informed consent, and written parental consent was provided for minors. These consenting participants were asked to contribute blood specimens at the initial enrollment visit and at scheduled appointments twice annually thereafter (ie, before and after the respiratory virus season). Beginning in 2017, post–influenza vaccination blood was collected from participants, and beginning in 2021, additional post–SARS-CoV-2 vaccination and prevaccination blood was collected. The most recent blood collected before the household infection/exposure was included and is referred to as the *preinfection/exposure specimen*. The most recent blood collected after the household infection/exposure is referred to as the *postinfection/exposure specimen*. The next-most recent blood collected after the postinfection/exposure specimen was included and referred to as the *extended postinfection/exposure specimen*.

### Measurement of IgG Antibodies Against Prefusion RSV F Protein

Sera were tested for anti–pre-F IgG antibodies with an electrochemiluminescence immunoassay (V-PLEX Respiratory Panel 1, IgG; Meso Scale Diagnostics). Serum samples were diluted 1:5000 in provided buffer and processed according to the manufacturer's protocol insert as previously described [16, 17]. Results were converted to binding arbitrary units (BAU) per milliliter and log_4_ transformed for analysis.

### Statistical Analysis

All individual- and household-level information was self-reported on enrollment and in annual reengagement surveys. Age, height, weight, and immunosuppression status were confirmed with data from electronic medical records (EMRs). RSV infection was defined by an RT-PCR–positive RSV test result for cases, and individuals residing in the household of a person with RSV infection were defined as RSV exposures. Participants enrolled in HIVE for more than 1 season who had multiple infection events in their household were included in this analysis. To be eligible, the infection event had to occur more than 1 year after the previous infection event, and case/household contact status was assigned independently for each infection event. Infection/exposure date was defined as the positive RT-PCR test result date for anyone infected in the household. Health rating was reported as self-perceived general health status on a visual analog scale of health (0, worst health imaginable; 100, best health imaginable). Body mass index was calculated by EMR-confirmed height and weight. Obesity was defined via the National Institutes of Health's predefined body mass index obesity cutoffs [18]. Immunosuppression status was assigned per person by the study team after collecting self-reported and EMR diagnoses.

Distributions of age, sex, race, day care, obesity, and immunosuppression status between cases and their household contacts were compared by χ^2^ and Fisher exact tests when appropriate. Differences in mean health rating were assessed with *t* tests. Means of untransformed IgG concentrations in cases and their household contacts were compared at all time points via Mann-Whitney *U* tests. Fold changes in geometric mean concentration (GMC) were calculated for cases and household contacts and compared with *t* tests. A fold rise in GMC ≥1.12 for cases with IgG increases pre- to postinfection was used as a cutoff for defining probable asymptomatic infections in exposed household contacts. Elapsed time between specimen collections and infections/exposures was calculated and compared with *t* tests.

The association between log_4_-transformed preinfection/exposure IgG concentration and RT-PCR–confirmed RSV infection was modeled by a Cox proportional hazards model adjusting for age. Adjustment variables were included on an a priori basis and if exposure and outcome associations had a *P* value ≤.05. Additionally, we modeled nonlinear changes in IgG concentrations over time using 2 generalized additive mixed models, with natural cubic splines applied to the time between specimen collection and infection/exposure date for cases and their household contacts, respectively. The first model included data from pre- and postinfection/exposure specimens, and the second model included data from postinfection to extended postinfection/exposure specimens. These models were used to predict IgG concentrations at set time points before and after infection/exposure for cases and household contacts. The predicted case and household contact values were compared by *z* tests at all prespecified time points. We performed statistical analyses in SAS software version 9.4 (SAS Institute Inc) and R software version 4.3.1 (R Software Inc). We considered a 2-sided *P* value ≤.05 to be statistically significant.

## RESULTS

Between 2014 and 2022, we included 422 individuals from 173 households with at least 1 positive RT-PCR test result for RSV out of the 3053 participants in the HIVE study. Of these, 113 (26.8%) were cases, 377 (89.3%) were household contacts, and 68 (16.1%) from households with more than 1 RSV infection in different seasons (>1 year apart) were treated as a case in 1 season and a household contact in another season. Of the 377 household contacts, 65 (17.2%) reported a qualifying illness at the time of RSV exposure and collected a study sample that was negative for RSV. The other 312 did not report a qualifying ARI, did not provide a sample, and were not tested. These individuals may have been asymptomatic or mildly symptomatic in a way that did not meet the case definition. The case and household contact characteristics were similar across seasons (Table 1). A majority of the study sample was 19 to 60 years old (69.2%), female (56.3%), and White (77.6%); did not attend day care (96.9%); and did not have obesity (89.4%). Individuals aged 0 to 12 years and those who attended day care were more likely to represent RSV cases, but the case to household contact distributions varied by season (Supplementary Table 1). The median household size was 4 (IQR, 4–5) and similar across seasons. The distributions of demographic, comorbidity, and household characteristics were similar when compared with all participants in the 2014–2021 HIVE cohort (data not shown). The median time between the preinfection/exposure specimen collection and infection/exposure was 60 days (IQR, 29–134); between infection/exposure and postinfection/exposure specimen collection, 155 days (IQR, 110–193); and between postinfection/exposure and extended postinfection/exposure specimen collection, 314 days (IQR, 275–342).

**Table 1.** Demographics and Comorbidities for Cases and Household Contacts

	All Seasons (n = 490), No. (%)	
	Cases (n = 113)	Household Contacts (n = 377)
Age, y		
0–12	37 (5.3)	49 (1.3)
13–18	14 (12.4)	37 (9.0)
19–60	61 (35.4)	280 (46.4)
≥61	1 (0.9)	11 (3.0)
Sex: female	67 (59.3)	209 (55.4)
Race		
White	90 (79.7)	290 (76.9)
Black or African American	9 (8.0)	22 (5.9)
Asian	10 (8.8)	28 (7.4)
Biracial or multiracial	0 (0.0)	6 (1.6)
American Indian or Alaska Native	0 (0.0)	2 (0.5)
Native Hawaiian or Pacific Islander	0 (0.0)	2 (0.5)
Other	4 (3.5)	27 (7.2)
Day care attendance	10 (8.6)	5 (1.3)
Health rating,^a^ median (IQR)	90 (80–95)	90 (80–95)
Obesity^b^	11 (9.7)	41 (10.9)
Immunosuppression diagnosis	3 (2.7)	2 (0.5)

Missing data for race, day care, and immunosuppressing medication were <10% and collapsed into the unexposed or other category.

^a^Health rating was self-reported on a continuous scale (100, best health you could imagine; 0, worst health you could imagine).

^b^Body mass index >30 indicated obesity.

**Figure 1.**
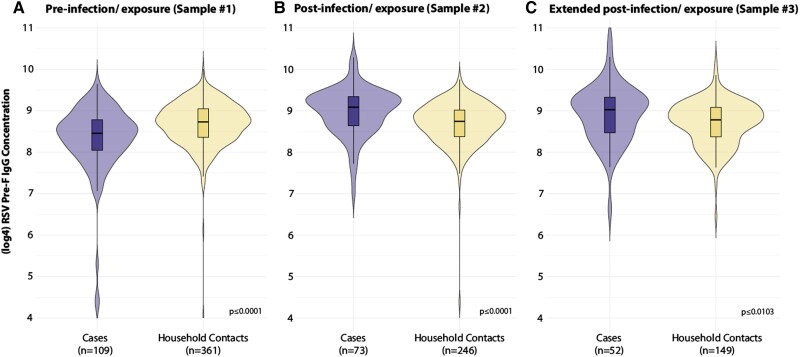
Log_4_-transformed RSV pre-F IgG for cases and household contacts preinfection/exposure, postinfection/exposure, and extended postinfection/exposure. Line = median; box = IQR; error bars = minimum and maximum; shading = distribution. IgG, immunoglobulin G; pre-F, prefusion F; RSV, respiratory syncytial virus.

We obtained preinfection/exposure IgG results for 109 (96.5%) cases and 361 (95.8%) household contacts, postinfection/exposure results for 73 (64.6%) cases and 246 (65.3%) household contacts, and extended postinfection/exposure results for 52 (46.0%) cases and 149 (38.7%) household contacts (Figure 1). At all sampling points (ie, preinfection, postinfection, and extended postinfection/exposure), we identified significant differences in median RSV pre-F IgG concentrations between cases and their household contacts. Cases had significantly lower preinfection IgG concentrations as compared with pre-exposure IgG concentrations in household contacts (median difference, 57 874 BAU/mL; *P* < .001) and significantly higher IgG concentrations postinfection and extended postinfection as compared with household contacts at the same time points (median differences: postinfection/exposure, 111 309 BAU/mL [*P* < .001]; extended postinfection/exposure, 77 916 [*P* < .001]; Figure 1).

Among those with increases between pre- and postinfection/exposure specimens, cases had a significantly greater rise in GMC than household contacts (GMC difference, 0.08; *P* = .001; Table 2). Of the 114 household contacts with pre- to post exposure IgG concentration increases, 8 (7.0%) had fold rises equal to or higher than the GMC rise in cases. Cases and household contacts with IgG increases showed a significant difference in time between postinfection and extended postinfection/exposure sample collections (median difference, 27 days; *P* = .05); no other groups or time windows between specimen collections exhibited significant differences. When examining the association between preinfection/exposure IgG concentration and RSV infection, we found that for every 1-unit increase in log_4_-transformed IgG concentration, there was a statistically significant 25% decrease in the hazard of RSV infection after adjusting for age (hazard ratio, 0.75; 95% CI, .63–.90; *P* < .002).

**Table 2.** Respiratory Syncytial Virus Pre-F IgG Concentrations for Cases and Their Household Contacts

	Cases (n = 113)		Household Contacts (n = 377)	
	No.	Median (IQR)	No.	Median (IQR)
**GMC fold rise**				
Specimen 1 to 2	58	1.12^a^	114	1.04^a^
Time from specimen 1 to household infection event, d		53 (31–79)		54 (23–98)
Time from household infection event to specimen 2, d		162 (119–186)		150 (120–196)
Specimen 2 to 3	14	1.04	68	1.04
Time from specimen 2 to 3, d		149 (112–174)^a^		176 (144–190)^a^
**GMC fold decrease**				
Specimen 1 to 2	15	0.97	132	0.97
Time from specimen 1 to household infection event, d		35 (19–89)		63 (29–140)
Time from household infection event to specimen 2, d		154 (127–205)		162 (112–194)
Specimen 2 to 3	38	0.97	81	0.97
Time from specimen 2 to 3, d		178 (152–192)		180 (158–195)

IgG concentrations are represented in binding arbitrary units per milliliter and were log transformed for GMC calculations. Variables were compared with the *t* test.

Abbreviations: GMC, geometric mean concentration; IgG, immunoglobulin G; pre-F, prefusion F.

^a^ *P* ≤ .05.

**Figure 2.**
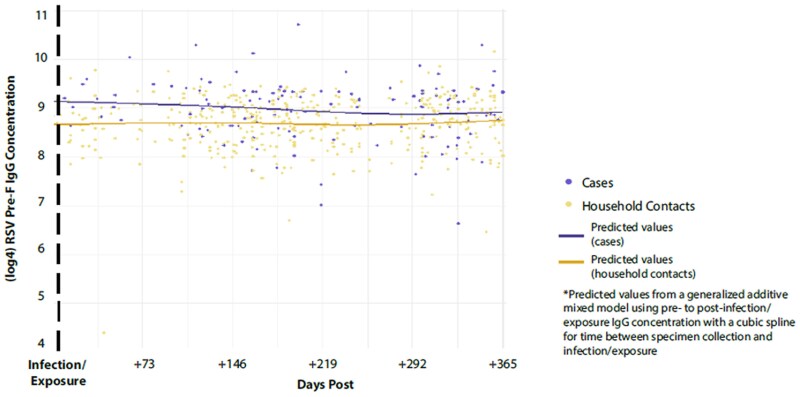
Log_4_-transformed RSV pre-F IgG for cases and household contacts across time with predicted values after infection/exposure from a generalized additive mixed model. IgG, immunoglobulin G; pre-F, prefusion F; RSV, respiratory syncytial virus.

After fitting a generalized additive mixed model investigating IgG concentrations over time, we were able to predict IgG concentrations at multiple time points before and after infection/exposure. Predicted values were lowest for cases 3 months before infection (log_4_ IgG concentrations [95% CI]: cases, 8.40 [8.13–8.68]; household contacts, 8.70 [8.62–8.78]; *P* = .05) and peaked in cases 1 month after infection (log_4_ IgG concentrations [95% CI]: cases, 9.12 [8.96–9.28]; household contacts, 8.67 [8.60–8.75]; *P* < .001; Table 3, Figure 2). After infection, predicted IgG concentrations were highest for cases as compared with their exposed household contacts at all investigated time points (predicted difference in log_4_ IgG concentrations: 1 month postinfection/exposure, 0.45; 3 months, 0.39; 6 months, 0.30; 1 year, 0.17). Predicted values were similar for household contacts at all time points. The only time points without statistically significant differences in predicted IgG concentrations were 1 month before infection/exposure and 1 year after infection/exposure.

**Table 3.** Predicted Levels of RSV Pre-F IgG Concentrations for Cases and Household Contacts Based on Generalized Additive Mixed Models

	Cases		Household Contact		
	IgG Concentration	95% CI	IgG Concentration	95% CI	*P* Value
Before infection/exposure					
3 mo	8.40	8.13–8.68	8.70	8.62–8.78	.05
1 mo	8.57	8.30–8.85	8.69	8.61–8.77	.45
After infection/exposure					
1 mo	9.12	8.96–9.28	8.67	8.60–8.75	<.001
3 mo	9.08	8.92–9.24	8.69	8.62–8.77	<.001
6 mo	8.98	8.82–9.14	8.68	8.61–8.75	<.001
1 y	8.92	8.76–9.08	8.75	8.68–8.82	.06

Two generalized additive mixed predictive models run before and after infection/exposure utilizing a cubic spline for time between specimen collection and infection/exposure date. Household contacts are inclusive of individuals with asymptomatic infections who were uninfected but exposed to RSV in their household. IgG concentrations are represented in log_4_-transformed binding arbitrary units per milliliter. Variables were compared with a *z* test.

Abbreviations: IgG, immunoglobulin G; pre-F, prefusion F; RSV, respiratory syncytial virus.

## DISCUSSION

Although RSV has been a focus of research for many decades, less is known regarding the dynamics of antibody responses resulting from infection with and protection against RSV, especially in those with mild or asymptomatic infection. When comparing those with infection and those with household exposure, we found that cases and household contacts had significantly different IgG concentrations at all 3 time points. We found the greatest boosts in pre-F IgG among cases. This supports the already widely accepted hypothesis that individuals produce pre-F IgG antibodies in response to RSV infection, while exposed persons can have pre-F IgG antibody increases of a lower magnitude from an immune response to RSV exposure [9, 19, 20]. Our predictive models allowed us to capture complex patterns in antibody levels, including initial boosts, plateaus, and subsequent declines over time, highlighting temporal differences in IgG concentrations observed between cases and household contacts. These models showed that boosts of pre-F IgG in cases were statistically different when compared with household contacts, peaked at 1 month after infection, and subsequently returned to baseline 1 year after infection. The pre- and postinfection/exposure IgG patterns predicted in the model were similar to a study during the COVID-19 pandemic that found a decrease to baseline antibody levels after just 1 year without RSV exposure [3].

When preinfection/exposure RSV pre-F IgG was used as a CoP accounting for age, we found that for each 1-unit increase in log_4_-transformed IgG, the hazard of RSV infection decreased by 25%. In a study of hospitalized patients, Piedra [20] found that a 1-unit increase in log_2_-transformed pre-F IgG concentration reduced the likelihood of RSV-associated hospitalization by 24.4%. However, this study used a different binding antibody laboratory assay for IgG detection and captured only severe RSV cases resulting in hospitalization. Our study collected specimens from individuals with a range of severity of illness, which could provide wider ranges of RSV IgG concentrations and correlates.

To truly understand seroprevalence, we must agree on a reproducible antibody threshold applicable to most individuals and populations. Some have used a 4-fold increase in antibody titer seropositivity threshold, as has been established for influenza [9]. We evaluated this by using the GMC IgG fold rise from pre- to postinfection in cases with IgG increases as an individual-level fold-rise cutoff in IgG concentration for seropositivity in this study. In our study, 7.0% of household contacts had a GMC IgG fold rise greater than the 1.12-fold rise identified in our cases, indicating likely asymptomatic cases. As prior studies have not been designed to capture the asymptomatic case population, it is difficult to determine whether this asymptomatic seroprevalence is consistent with other findings. Of the few available studies, the percentage of asymptomatic RSV cases ranged from 1% to 42% [4, 21, 22]. However, these studies used vastly different populations from multiple countries, as well as unique sampling methods, which could have led to inconsistent results. For example, in contrast to our symptom-triggered sampling of the household, the study with the highest detection rate (42%) collected swabs on a weekly basis, which increases the ability to detect asymptomatic infections [4]. Although weekly swabbing will likely capture more asymptomatic cases, it is not always time- or cost-effective. Therefore, to better understand the incidence of asymptomatic cases in studies without the ability to complete weekly sampling, we can use change in IgG thresholds to identify asymptomatic cases.

Our study has multiple strengths, including a standardized CoP assay, multiple years of cohort data, and the ability to document the timing of RSV exposure among household contacts of people infected with RSV. The HIVE cohort has over a decade of data and specimens, which allowed us to investigate this question among household populations. Due to their interactions with other children in childcare and social settings, children are often exposed to and infected with RSV, which they can then bring back to the household. This gives us the opportunity to look at RSV infections among not only children but also adults. Additionally, in HIVE, we have high certainty that all household contacts of individuals with RSV are exposed. For example, in this cohort, illness reporting occurs via weekly phone call or email check-ins with the study team, where the household reporter details any ARI symptoms exhibited by each member of the household. Last, the active incentivized illness reporting used by the HIVE cohort reduces missed detection of respiratory virus infections in these households.

Our study is not without limitations. First, we cannot ensure that all RSV infections were captured by RT-PCR. To accurately detect all infections, a person would need to be repeatedly sampled and tested for respiratory pathogens on a weekly, even daily, basis [4, 21–23]. Additionally, individuals in this cohort are motivated to participate in weekly symptom reports and multiple blood draws each season and therefore may not be generalizable with the average American adult. This is a common limitation in cohort studies, which are more likely to recruit people motivated by outside factors, such as more health-conscious behavior and interest in scientific research. Although participation in this cohort is incentivized, we cannot control or fully understand the motivations of our study participants and thus must acknowledge this as a limitation. Last, due to our inclusion criteria, we cannot rule out community-acquired RSV infections in these households, but future studies with routine asymptomatic sampling could address this issue.

We found that lower concentrations of RSV pre-F IgG increase the susceptibility of RSV infection for mild to moderate illnesses in community settings. These less severe illnesses can still result in substantial burden for those who are younger and attending day care [24, 25]. To reduce the risk of RSV infection in these individuals, we must prioritize them to receive RSV vaccinations and immunizations that are available, and we must work toward new therapies that boost pre-F IgG levels in persons without available preventions, such as nonelderly adults and nonpregnant persons. Additionally, we conclude that RSV pre-F IgG–mediated protection derived from RSV infection starts to wane 6 months after the infection event. Therefore, timing of the administration of RSV immunologic preventive measures should be closely evaluated to ensure that people with the highest risk of severe disease are protected throughout the RSV season.

## Supplementary Material

jiaf168_Supplementary_Data

## Supplementary Data

Supplementary materials are available at *The Journal of Infectious Diseases* online (http://jid.oxfordjournals.org/). Supplementary materials consist of data provided by the author that are published to benefit the reader. The posted materials are not copyedited. The contents of all supplementary data are the sole responsibility of the authors. Questions or messages regarding errors should be addressed to the author.

## Notes

***Acknowledgments.*** We thank all individuals for their participation in the HIVE study.

***Author contributions.*** K. E. R.: conceptualization, formal analysis, investigation, methodology, software, validation, visualization, writing–original draft preparation, writing–reviewing and editing. C. J.: investigation, validation, writing–reviewing and editing. M. S.: investigation, software, writing–reviewing and editing. A. C.: investigation, software, writing–reviewing and editing. Y. Y.: methodology, software, writing–reviewing and editing. A. S. M.: funding acquisition, resources, supervision, writing–reviewing and editing. A. S. L.: conceptualization, project administration, supervision, writing–reviewing and editing. E. T. M.: conceptualization, funding acquisition, project administration, resources, supervision, writing–reviewing and editing.

***Financial support.*** This work was supported by federal funds from the National Institutes of Health (HSN272201400005C to A. S. M.; 75N93021C00015 to A. S. M. and E. M.); and the Centers for Disease Control and Prevention (U01IP000474, 1R01A1097150, R56A1097150, U01IP001034 to A. S. M.).
