# Supplementary material for: Strength and Durability of Respiratory Syncytial Virus Prefusion F Immunoglobulin G Following Infection and Exposure in a Household Cohort, 2014–2022
Source: J Infect Dis. 2025 Apr 3;231(6):e1138–45. doi: 10.1093/infdis/jiaf168 (PMC12247821; doi:10.1093/infdis/jiaf168)
Supplement: jiaf168_Supplementary_Data [file jiaf168_supplementary_data.docx]

**Supplemental Table 1**

| **Supplemental Table 1.** Demographics and comorbidities for cases and their household contacts by study season | | | | | | | | | | | | | | |
| --- | --- | --- | --- | --- | --- | --- | --- | --- | --- | --- | --- | --- | --- | --- |
|  | **2014-2015**  **(n=65)** | | **2015-2016**  **(n=52)** | | **2016-2017**  **(n=78)** | | **2017-2018**  **(n=93)** | | **2018-2019**  **(n=93)** | | **2019-2020**  **(n=110)** | | **2021-2022**  **(n=20)** | |
|  | **C**  **n=11** | **H**  **n=54** | **C**  **n=10** | **H**  **n=42** | **C**  **n=16** | **H**  **n=41** | **C**  **n=22** | **H**  **n=71** | **C**  **n=22** | **H**  **n=71** | **C**  **n=28** | **H**  **n=82** | **C**  **n=4** | **H**  **n=16** |
| **Age,** n(%) |  |  |  |  |  |  |  |  |  |  |  |  |  |  |
| 0-2 years | 0  (0.0) | 0  (0.0) | 0  (0.0) | 0  (0.0) | 0  (0.0) | 0  (0.0) | 0  (0.0) | 3  (4.2) | 3  (13.6) | 0  (0.0) | 3  (10.7) | 2  (2.4) | 0  (0.0) | 0  (0.0) |
| 3-12 years | 0  (0.0) | 0  (0.0) | 0  (0.0) | 0  (0.0) | 3  (18.8) | 4  (9.8) | 7  (31.8) | 9  (12.7) | 6  (27.3) | 20  (28.2) | 15  (53.6) | 14  (17.1) | 0  (0.0) | 2  (12.5) |
| 13-18 years | 1  (9.1) | 7  (13.0) | 1  (10.0) | 6  (14.3) | 1  (6.2) | 5  (12.2) | 5  (22.7) | 6  (8.5) | 3  (13.6) | 3  (4.2) | 1  (3.6) | 6  (7.3) | 2  (50.0) | 1  (6.3) |
| 19-40 years | 5  (45.4) | 26  (48.1) | 5  (50.0) | 14  (33.3) | 5  (31.2) | 17  (41.4) | 7  (31.8) | 29  (40.8) | 8  (36.4) | 32  (45.1) | 8  (28.5) | 49  (59.8) | 2  (50.0) | 8  (50.0) |
| 41-60 years | 4  (36.4) | 19  (35.2) | 4  (40.0) | 19  (45.2) | 7  (43.8) | 11  (26.8) | 3  (13.7) | 23  (32.4) | 2  (9.1) | 15  (21.1) | 1  (3.6) | 11  (13.4) | 0  (0.0) | 5  (31.2) |
| 61+ years | 1  (9.1) | 2  (3.7) | 0  (0.0) | 3  (7.2) | 0  (0.0) | 4  (9.8) | 0  (0.0) | 1  (1.4) | 0  (0.0) | 1  (1.4) | 0  (0.0) | 0  (0.0) | 0  (0.0) | 0  (0.0) |
| **Sex, female,** n(%) | 7 (63.6) | 34 (63.0) | 7  (70.0) | 20  (47.6) | 9 (56.3) | 22 (53.7) | 14 (63.6) | 41  (57.8) | 14  (63.6) | 35 (49.3) | 13  (46.4) | 45  (54.9) | 3 (75.0) | 12 (75.0) |
| **Race,** n(%) |  |  |  |  |  |  |  |  |  |  |  |  |  |  |
| White | 6  (54.6) | 42  (77.8) | 5  (50.0) | 26  (61.9) | 13  (81.3) | 33  (80.5) | 20  (90.9) | 53  (74.7) | 19  (86.4) | 60  (84.5) | 23  (82.1) | 60  (73.1) | 4  (100) | 16  (100) |
| Black or African American | 3  (27.2) | 4  (7.4) | 1  (10.0) | 5  (11.9) | 1  (6.2) | 3  (7.3) | 0  (0.0) | 2  (2.8) | 3  (13.6) | 4  (5.7) | 1  (3.6) | 4  (4.9) | 0 (0.0) | 0 (0.0) |
| Asian | 1  (9.1) | 2  (3.7) | 4  (40.0) | 6  (14.3) | 2  (12.5) | 4  (9.8) | 0  (0.0) | 4  (5.6) | 0 (0.0) | 1  (1.4) | 3  (10.7) | 11  (13.4) | 0  (0.0) | 0  (0.0) |
| Biracial or Multiracial | 0  (0.0) | 0  (0.0) | 0  (0.0) | 0  (0.0) | 0 (0.0) | 0  (0.0) | 0 (0.0) | 3  (4.2) | 0 (0.0) | 0 (0.0) | 0 (0.0) | 3  (3.7) | 0 (0.0) | 0 (0.0) |
| American Indian or Alaska Native | 0  (0.0) | 0  (0.0) | 0  (0.0) | 0  (0.0) | 0 (0.0) | 0 (0.0) | 0 (0.0) | 1  (1.4) | 0 (0.0) | 1  (1.4) | 0 (0.0) | 0 (0.0) | 0 (0.0) | 0 (0.0) |
| Native Hawaiian or Pacific Islander | 0  (0.0) | 0  (0.0) | 0  (0.0) | 1  (2.4) | 0 (0.0) | 1  (2.4) | 0 (0.0) | 0 (0.0) | 0 (0.0) | 0 (0.0) | 0 (0.0) | 0 (0.0) | 0 (0.0) | 0 (0.0) |
| Other | 1  (9.1) | 6  (11.1) | 0  (0.0) | 4  (9.5) | 0 (0.0) | 0 (0.0) | 2  (9.1) | 8  (11.3) | 0 (0.0) | 5  (7.0) | 1  (3.6) | 4  (4.9) | 0 (0.0) | 0 (0.0) |
| **Daycare,** n(%) | 0 (0.0) | 0 (0.0) | 0 (0.0) | 0 (0.0) | 1  (6.3) | 0 (0.0) | 0  (0.0) | 1  (1.4) | 3  (13.6) | 1  (1.4) | 6  (21.4) | 3  (3.7) | 0 (0.0) | 0 (0.0) |
| **Health Rating,** median(IQR) | 80 (65, 95) | 90 (85, 95) | 90 (85, 92) | 90 (85, 95) | 90 (85, 95) | 90 (80, 91) | 90 (90, 95) | 90 (80, 95) | 90 (80, 95) | 90 (85, 95) | 88 (80, 95) | 85 (80, 90) | 83 (75, 85) | 77 (70, 88) |
| **Obesity,** n(%) | 1  (9.1) | 9  (16.7) | 3  (30.0) | 2  (4.8) | 0  (0.0) | 3  (7.3) | 1  (4.6) | 8  (11.3) | 1  (4.6) | 6  (8.5) | 4  (14.3) | 10  (12.2) | 1  (25.0) | 3  (18.8) |
| **Immunosupp. medications,** n(%) | 0 (0.0) | 0 (0.0) | 0 (0.0) | 0 (0.0) | 1  (6.3) | 0 (0.0) | 2  (9.1) | 0 (0.0) | 0 (0.0) | 1  (1.4) | 0 (0.0) | 1  (1.2) | 0 (0.0) | 0 (0.0) |
| Notes: median (interquartile range) or n(%); race, daycare, and immunosuppressing medication missing data was <10% and collapsed into the unexposed or other category; Daycare represents the number of individuals attending daycare, Health rating was self-reported on a continuous scale with 100 representing the “best health you could imagine” and 0 representing the “worst health you could imagine”, Body mass index (BMI) greater than 30 indicated obesity  Abbreviations: BMI = body mass index; Immunosupp = Immunosuppressing | | | | | | | | | | | | | | |
